# Trends in the Use and Indications for Intracytoplasmic Sperm Injection Between 2005 and 2017: A State‐Wide Descriptive Cohort Analysis

**DOI:** 10.1111/ajo.70070

**Published:** 2025-11-05

**Authors:** Aleah Kink, Parinaz Mehdipour, Richard J. Hiscock, Beverley J. Vollenhoven, Catharyn J. Stern, Susan P. Walker, Mark P. Green, Tiki Osianlis, Franca Agresta, David Wilkinson, Stephen Tong, Roxanne Hastie, Amber L. Kennedy, Anthea C. Lindquist

**Affiliations:** ^1^ Melbourne Medical School The University of Melbourne Parkville Victoria Australia; ^2^ Department of Obstetrics, Gynaecology and Newborn Health The University of Melbourne Parkville Victoria Australia; ^3^ Department of Obstetrics and Gynaecology Monash University Clayton Victoria Australia; ^4^ Monash IVF Clayton Victoria Australia; ^5^ Reproductive Services Unit The Royal Women's Hospital Parkville Victoria Australia; ^6^ Melbourne IVF East Melbourne Victoria Australia; ^7^ Mercy Perinatal, Mercy Hospital for Women Heidelberg Victoria Australia; ^8^ School of BioSciences The University of Melbourne Parkville Victoria Australia; ^9^ Newlife IVF East Melbourne Victoria Australia; ^10^ City Fertility Centre Melbourne Victoria Australia

**Keywords:** fertilisation in vitro, fertility, infertility, reproductive techniques (assisted), sperm injections (intracytoplasmic)

## Abstract

**Background:**

Intracytoplasmic sperm injection (ICSI) was first developed to overcome male factor infertility. ICSI has increased in uptake globally, including in cases where its use is non‐essential for fertilisation.

**Aims:**

To identify temporal trends in the use of, and indications for ICSI in an Australian context.

**Materials and Methods:**

A statewide descriptive cohort study examining the trends in ICSI uptake and reported indication/s for ICSI use. The cohort included women undergoing IVF between 2005 and 2017 at IVF clinics across Victoria, Australia that resulted in a birth after 20 weeks' gestation.

**Results:**

The dataset comprised 32 102 assisted reproduction cycles: 22 873 (71.3%) ICSI and 9229 (28.7%) conventional IVF. In 2005, ICSI accounted for 60.6% (1182/1952) of cycles, increasing to 79.5% (2344/2947) by 2017 (*p*_trend_ < 0.001). Testicular sperm retrieval as an indication for ICSI remained consistent over time (*p*_trend_ = 0.15). Male factor infertility as an indication decreased over time (*p*_trend_ = 0.007). Vitrified oocyte thaw (*p*_trend_ = 0.016) and ‘unexplained subfertility’ (*p*_trend_ = 0.30) cycles did not surpass 1.7% (39/2293) and 0.4% (9/2048), respectively of total cycles in any year. Donor sperm (*p*_trend_ = 0.001), pre‐implantation genetic testing (*p*_trend_ = 0.004), female factors associated with poor IVF outcome (*p*_trend_ = 0.005) and advanced maternal age (*p*_trend_ = 0.005) all increased as indications for ICSI over time. ‘Unspecified’ indication accounted for the majority of ICSI cycles after 2008 (*p*_trend_ = 0.015).

**Conclusions:**

During our study period, the total use of ICSI increased by 18.9%. Notably, most of these cycles were not medically indicated.

## 1 Introduction

Intracytoplasmic sperm injection (ICSI) is an assisted reproductive technology, first introduced clinically in 1992 [1]. ICSI involves retrieval of sperm from an ejaculated or surgically obtained sample; a single spermatozoon is then injected directly into the cytoplasm of a mature oocyte during an in vitro fertilisation (IVF) cycle [2].

ICSI was developed to overcome male factor infertility, or fertilisation failure during conventional IVF [3]. It is now used in the context of thawed oocytes, where the vitrification and thawing process results in hardening of the zona pellucida, impeding the natural sperm penetration required in conventional IVF [4]. However, ICSI is increasingly used for indications where the evidence is less clear—low oocyte count (≤ 6) [5], pre‐implantation genetic testing for aneuploidy (PGT‐A) [6], oocytes that have undergone in vitro maturation (IVM) [7], unexplained infertility [8], poor quality oocytes [9], advanced maternal age (≥ 40 years old) [10], use of donor sperm and in human immunodeficiency virus (HIV) or hepatitis discordant couples [11].

In the absence of male factor infertility, evidence is limited to support the use of ICSI in improving fertilisation rate, embryo quality and number, implantation success or live birth rate [12, 13, 14, 15, 16, 17] and may increase the risk of birth defects when compared with conventional IVF [18]. Nevertheless, there continues to be an increase in the global number of assisted reproduction cycles [19], with a surge in the uptake of ICSI for non‐male factor infertility [20]. Some fertility centres now use ICSI in all IVF cycles [12].

There are limited studies examining trends in use and indications for ICSI. Should ICSI be associated with adverse childhood outcomes, then understanding both the magnitude and indications for use of ICSI is critical. Evidence of increased use of ICSI for non‐essential indications would support the need for further research into pregnancy, birth and childhood outcomes following ICSI conception compared with conventional IVF. Utilising a large Victorian cohort, our study aimed to examine the temporal trends in use and indications for ICSI between 2005 and 2017.

## 2 Materials and Methods

We performed a descriptive cohort study of all assisted reproduction cycles initiated between 2005 and 2017 from the three major IVF providers in Victoria during the study era: Melbourne IVF, Monash IVF and City Fertility Centre. Reported indications for the use of ICSI over conventional IVF were examined.

The study population included all ICSI or conventional IVF cycles occurring between 2005 and 2017 that resulted in a birth after 20 weeks' gestation (inclusive of multiple births, congenital anomalies, terminations and stillbirths). We excluded cycles if the cycle year or fertilisation method could not be determined, and cycles where half the oocytes were inseminated via ICSI, and the other half via conventional IVF. The dataset included patient demographics, cycle characteristics, embryo quality, pregnancy and birth outcomes.

Demographic data were extracted for patient age, region of birth, gravidity, parity, partner gender, number of miscarriages and number of prior IVF stimulation cycles. As the IVF databases did not define specific indications for ICSI or conventional IVF cycles, indication data were indirectly assessed by collating information from free text fields and other relevant variables (sperm collection method, sperm source, semen parameters and oocyte yield), with final indications determined by expert consensus. Where more than one indication for ICSI was reported, a single ‘most applicable’ indication was applied as per the following hierarchical order: testicular sperm retrieval, male factor infertility, vitrified oocyte thaw cycle, donor sperm, female factors associated with poor IVF outcomes, advanced maternal age (≥ 40 years old), pre‐implantation genetic testing, ‘unexplained subfertility’ and ‘unspecified’ [9]. Clear indications for ICSI were considered those in which ICSI is essential for fertilisation such as in male factor fertility and testicular sperm retrieval. There is also evidence supporting ICSI use for vitrified oocyte thaw cycles and previous fertilisation failure with conventional IVF [3, 4]. Less clear indications were considered those where ICSI is not definitively required for successful fertilisation such as cases of donor sperm, female factors associated with poor IVF outcomes, advanced maternal age (≥ 40 years old), pre‐implantation genetic testing, ‘unexplained subfertility’ and ‘unspecified’.

Male factor infertility included pre‐diagnosis of oligoasthenoteratozoospermia, total motile sperm count < 2 × 10^6^ post sperm preparation on the day of a planned IVF cycle, and other causes of male infertility such as sperm antibodies, chromosomal defects, and not otherwise specified. Testicular sperm retrieval assumed open or needle biopsy sperm retrieval. Female factors associated with poor IVF outcomes included previous failed conventional IVF cycle/s, poor ovarian response [21], low oocyte count (≤ 6), premature or occult ovarian failure, oncological fertility preservation, or post‐menopause. Pre‐implantation genetic testing included cycles where either genetic testing was planned or did occur. ‘Unspecified’ indication included cycles where an appropriate indication was not evident from the available data, including general indications for assisted reproduction, but not for ICSI such as tubal defects, polycystic ovaries, ovulation defects, endometriosis, genetic disorders, uterine polyps and/or fibroids.

Due to data collection and recording differences between the IVF labs, there was inconsistency in the availability of extracted data. Data were missing for some demographic variables, as well as those used to determine ICSI indication. The proportion of missing demographic data were examined.

Descriptive statistics were calculated, with frequency and proportion reported for all categorical variables. Analysis of variance was used to assess trends over time for categorical variables. The median and interquartile range were reported for continuous variables with a normal distribution. Baseline patient characteristics were provided for different cycle types. Pearson chi‐square tests were used to compare characteristics between ICSI and conventional IVF cohorts and between cohort years 2005 and 2017. Results are presented as % difference, with associated 95% confidence limits and *p*‐values.

Statistical analyses were conducted in Stata/BE (Version 18.0, College Station, TX, USA). All figures were constructed with GraphPad Prism (Version 10.0.1 (170)) and Microsoft PowerPoint (Version 16.75).

Informed consent was not required due to the retrospective nature of the study; all patient data were de‐identified, and a waiver of consent was provided by the Human Research Ethics Committee. Ethical approval for the creation of the dataset was obtained from the Mercy Health, Monash Health and Melbourne IVF Health Human Research Ethics Committees [22]. Amended ethical approval, specific to this study, was granted by the Mercy Health Human Research Ethics Committee (2018‐017).

## 3 Results

**FIGURE 1:**
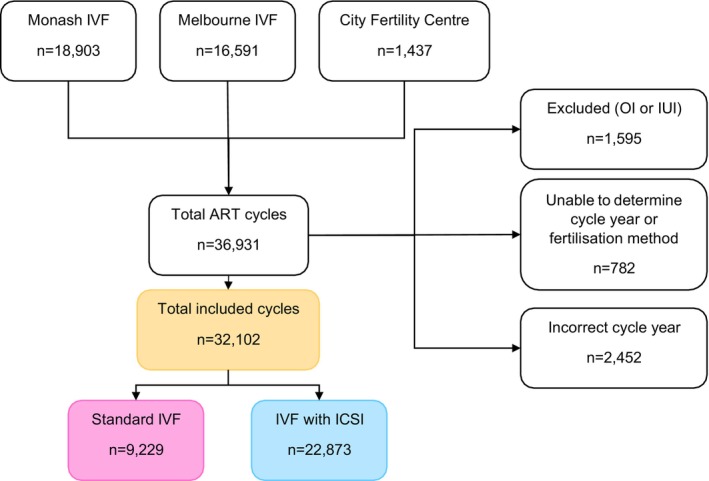
Study inclusion flow‐chart. ART, assisted reproductive technology; ICSI, intracytoplasmic sperm injection; IUI, intrauterine insemination; IVF, in vitro fertilisation; OI, ovulation induction.

Our cohort included 36 931 assisted reproduction cycles. 4829 cycles were excluded as they were an ovulation induction or intrauterine insemination cycle (*n* = 1595), fertilisation method or cycle year could not be determined due to missing data (*n* = 782), or the cycle year was not between 2005 and 2017 (*n* = 2452) (Figure 1). The remaining 32 102 cycles included 22 873 (71.3%) ICSI and 9229 (28.7%) conventional IVF cycles.

The median age among the overall study cohort was 34.7 years (interquartile range: 31.7–37.8; range: 20–53 years) (Table 1). Patient age data were missing for 14.2% (*n* = 4569).

**TABLE 1:** Patient characteristics for conventional IVF, ICSI and total cycles, 2005–2017.

Demographics	IVF, *n* (%)	ICSI, *n* (%)	Total, *n* (%)
	*N* = 9229	*N* = 22 873	*N* = 32 102
*Patient age*			
Median (IQR)	34.6 (31.7–37.3)	34.8 (31.7–37.9)	34.7 (31.7–37.8)
< 25 years	53 (0.6)	269 (1.2)	322 (1.0)
25–29 years	934 (10.1)	2621 (11.5)	3555 (11.1)
30–34 years	2987 (32.4)	7547 (33.0)	10 534 (32.8)
35–39 years	2676 (29.0)	7307 (31.9)	9983 (31.1)
≥ 40 years	664 (7.2)	2475 (10.8)	3139 (9.8)
Missing	1915 (20.7)	2654 (11.6)	4569 (14.2)
*Partner gender*			
Male	7339 (79.5)	18 875 (82.5)	26 214 (81.7)
Female	8 (0.1)	694 (3.0)	702 (2.2)
Missing or no partner	1882 (20.4)	3304 (14.4)	5186 (16.2)
*Maternal region of birth*			
Oceania and Antarctica^a^	1017 (11.0)	4584 (20.0)	5601 (17.4)
North‐West Europe	80 (0.9)	283 (1.2)	363 (1.1)
Southern and Eastern Europe	39 (0.4)	147 (0.6)	186 (0.6)
North Africa and the Middle East	13 (0.1)	77 (0.3)	90 (0.3)
South‐East Asia	79 (0.9)	292 (1.3)	371 (1.2)
North‐East Asia	86 (0.9)	263 (1.1)	349 (1.1)
Southern and Central Asia	124 (1.3)	366 (1.6)	490 (1.5)
Americas	26 (0.3)	83 (0.4)	109 (0.3)
Sub‐Saharan Africa	26 (0.3)	90 (0.4)	116 (0.4)
Missing	7739 (83.9)	16 688 (73.0)	24 427 (76.1)
*Gravidity*			
0	2005 (21.7)	5886 (25.7)	7891 (24.6)
1	1419 (15.4)	4068 (17.8)	5487 (17.1)
2	596 (6.5)	1600 (7.0)	2196 (6.8)
≥ 3	515 (5.6)	1169 (5.1)	1684 (5.2)
Missing	4694 (50.9)	10 150 (44.4)	14 844 (46.2)
*Parity*			
0	5251 (56.9)	14 448 (63.2)	19 699 (61.4)
1	1861 (20.2)	5287 (23.1)	7148 (22.3)
2	167 (1.8)	465 (2.0)	632 (2.0)
≥ 3	54 (0.6)	154 (0.7)	208 (0.6)
Missing	1896 (20.5)	2519 (11.0)	4415 (13.8)
*Number of miscarriages*			
0	4180 (45.3)	11 101 (48.5)	15 281 (47.6)
1	436 (4.7)	1566 (6.8)	2002 (6.2)
2	69 (0.7)	251 (1.1)	320 (1.0)
≥ 3	14 (0.2)	80 (0.3)	94 (0.3)
Missing	4530 (49.1)	9875 (43.2)	14 405 (44.9)
*Number of prior IVF stimulation cycles*			
0	1791 (19.4)	3293 (14.4)	5084 (15.8)
1	3621 (39.2)	8094 (35.4)	11 715 (36.5)
2	1006 (11.0)	3997 (17.5)	5003 (15.6)
≥ 3	759 (8.2)	4720 (20.6)	5479 (17.1)
Missing	2052 (22.2)	2769 (12.1)	4821 (15.0)

Abbreviations: ICSI, intracytoplasmic sperm injection; IQR, interquartile range; IVF, in vitro fertilisation.

^a^ Of which IVF = 978 (10.6), ICSI = 4440 (19.4) and total = 5418 (16.9) were born in Australia.

Among the study cohort, 81.7% of patients (*n* = 26 214) had a male partner at the time of the cycle. Among same‐sex couples, 98.9% (694/702) underwent ICSI, with 1.1% (8/702) undergoing conventional IVF.

There was a high proportion of data missing for gravidity (46.2%, *n* = 14 844), parity (13.8%, *n* = 4415) and miscarriage (44.9%, *n* = 14 405). Based on available data, most patients were nulliparous (61.4%, *n* = 19 699) and 7.5% (*n* = 2416) of the total cohort had a documented previous miscarriage.

Patients undergoing an ICSI cycle had a significantly higher number of prior conventional IVF stimulation cycles, with 20.6% (4720/22 873) of ICSI patients having had three or more prior cycles compared with 8.2% (759/9229) of conventional IVF cycle patients.

**FIGURE 2:**
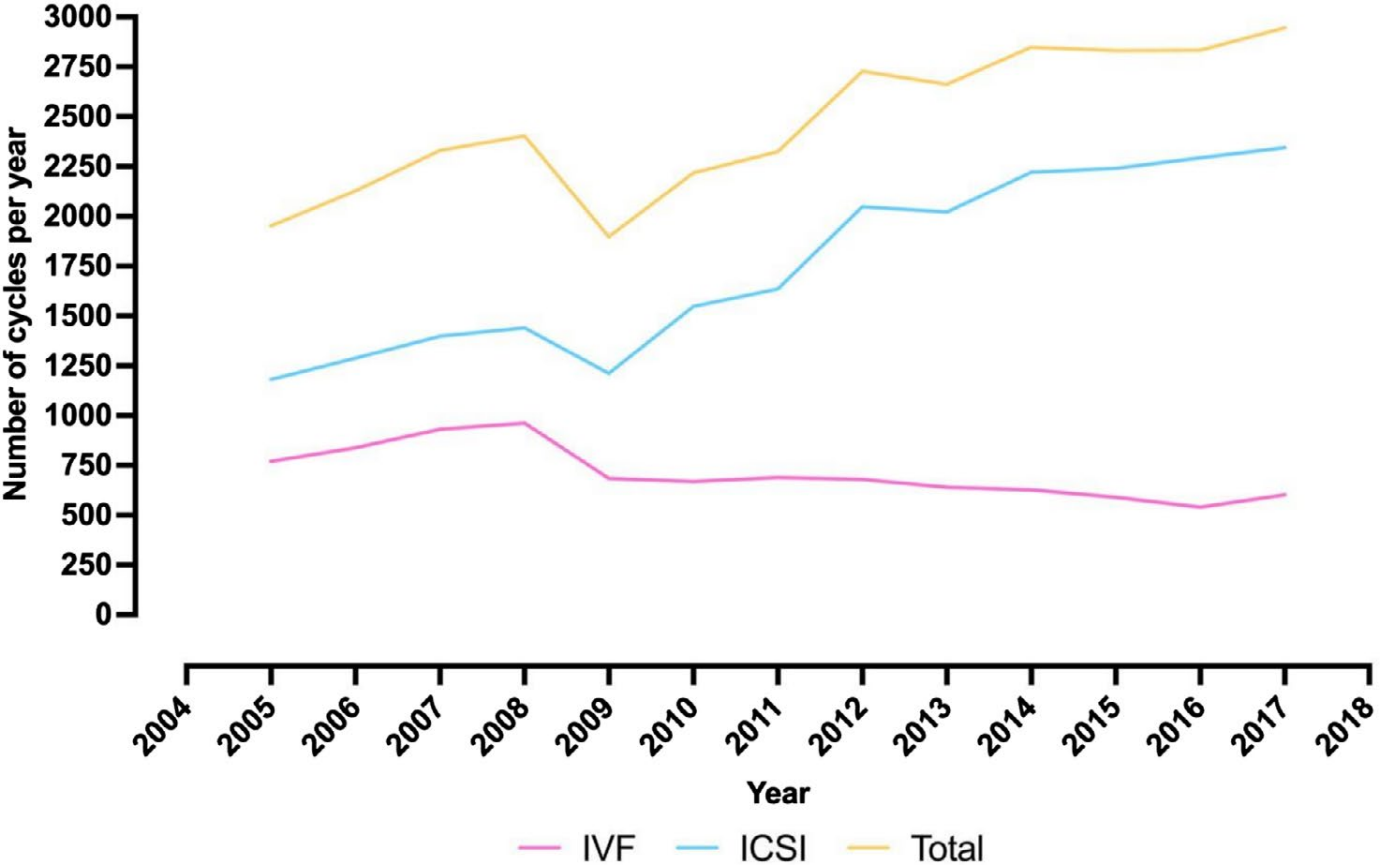
Line chart displaying annual number of conventional IVF, ICSI and total cycles, 2005–2017. IVF, in vitro fertilisation; ICSI, intracytoplasmic sperm injection.

There was a significant change in the proportion of ICSI cycles compared to conventional IVF cycles between 2005 and 2017 (Figure 2, Table S1). In 2005, ICSI accounted for 60.6% (1182/1952) of total cycles which increased to 79.5% (2344/2947) by 2017 (difference 18.9% (95% CI: 16.4–21.6), *p*_trend_ < 0.001) (Figure S1). The proportion of conventional IVF cycles peaked in 2008, at 40.1% (963/2404). In 2009, there was a notable reduction in the total number of cycles. However, after this period, the proportion of ICSI cycles increased with each year, and the number of conventional IVF cycles decreased.

**FIGURE 3:**
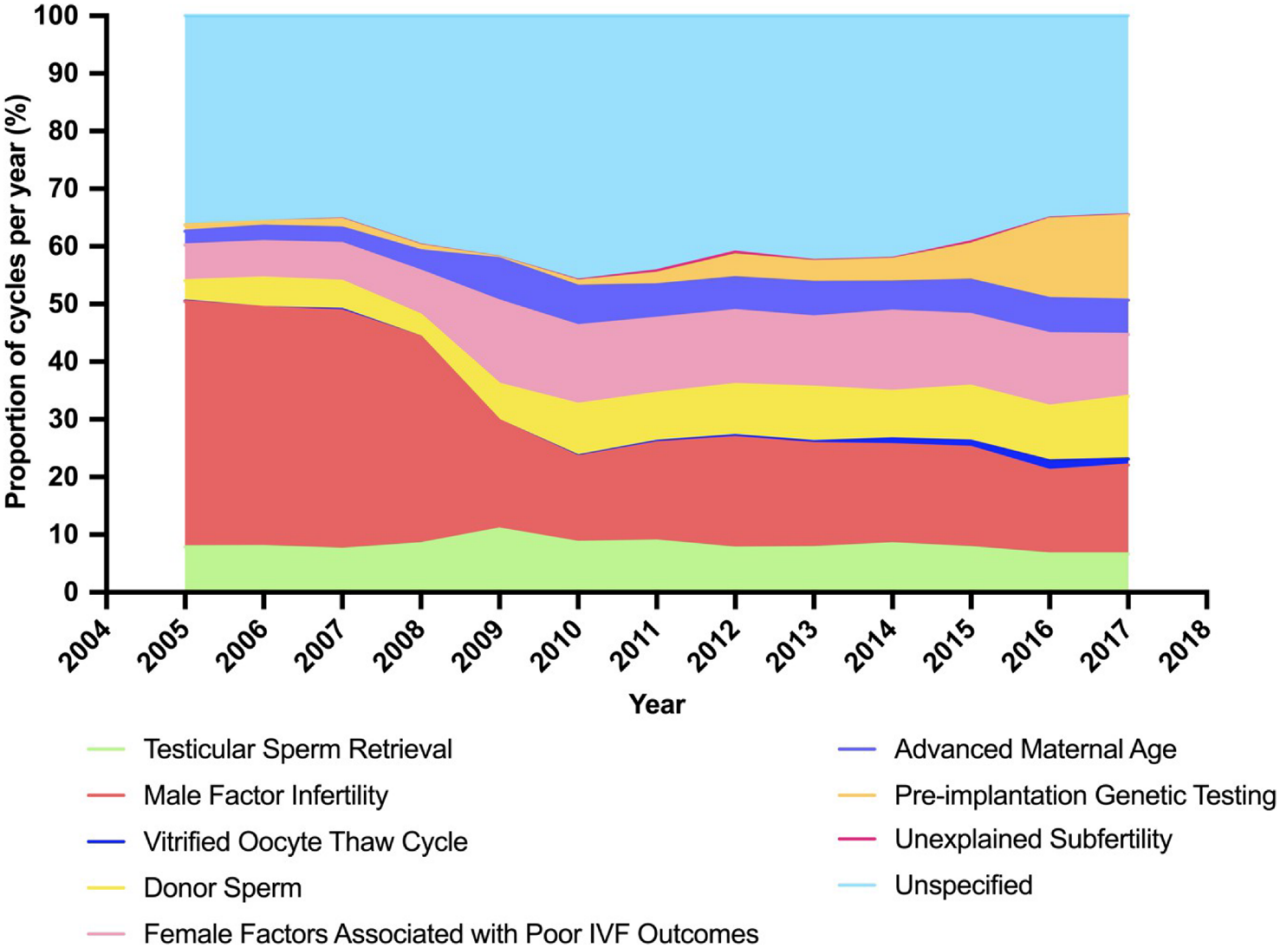
Stacked area chart displaying the proportion of ICSI cycles, stratified by indication for ICSI, 2005–2017. ICSI, intracytoplasmic sperm injection; IVF, in vitro fertilisation. Testicular sperm retrieval: *p*_trend_ = 0.15; male factor infertility: *p*_trend_ = 0.007; vitrified oocyte thaw cycle: *p*_trend_ = 0.016; donor sperm: *p*_trend_ = 0.001; female factors associated with poor IVF outcomes: *p*_trend_ = 0.005; advanced maternal age: *p*_trend_ = 0.005; pre‐implantation genetic testing: *p*_trend_ = 0.004; unexplained subfertility: *p*_trend_ = 0.30, unspecified: *p*_trend_ = 0.015.

Testicular sperm retrieval, a universal indication for ICSI, remained consistent over time, accounting for 7.9% (93/1182) of cycles in 2005, and 6.7% (156/2344) by 2017 (*p*_trend_ = 0.15, difference −1.2% (95% CI: −3.0 to 0.6)) (Figure 3, Table S2, Figure S2). Male factor infertility was the primary reported indication for ICSI prior to 2008 but decreased over time (*p*_trend_ = 0.007). The number of cycles with an ‘unspecified’ indication increased over time (*p*_trend_ = 0.015), accounting for up to 45.9% (710/1548) of cycles by 2010.

The number of vitrified oocyte thaw cycles, as an indication for ICSI, did not surpass 1.7% (39/2293) of total cycles in any calendar year (*p*_trend_ = 0.016). Cycles where the diagnosis was recorded as ‘unexplained subfertility’, accounted for no more than 0.4% (9/2048) of cycles in any given year (*p*_trend_ = 0.30). The use of donor sperm as an indication for ICSI increased significantly during the study period, accounting for 3.6% (42/1182) of cycles in 2005, and 10.9% (255/2344) of cycles by 2017 (*p*_trend_ = 0.001, difference 7.3% (95% CI: 5.7–9.0)).

Female factors associated with poor IVF outcomes increased significantly as an indication for ICSI during the study period (*p*_trend_ = 0.005), as did advanced maternal age (*p*_trend_ = 0.005). Pre‐implantation genetic testing as an indication for ICSI rose from 0.9% (14/1548) of cycles in 2010 to 14.7% (344/2344) of cycles by 2017 (*p*_trend_ = 0.004, difference 13.8% (95% CI: 12.2–15.3)).

## 4 Discussion

Our large Australian study examines the temporal changes in utilisation and indications for ICSI in Victoria between 2005 and 2017. Our findings suggest an increase in uptake of ICSI over conventional IVF with each year, such that the uptake of ICSI now outweighs conventional IVF. In 2005, ICSI accounted for 60.6% of cycles, increasing to 79.5% by 2017. Testicular sperm retrieval as an indication for ICSI remained consistent over time. However, the use of ICSI in the absence of clear, reported indications has increased since 2009, now accounting for the majority of ICSI cycles. This reflects international trends which show a continued increase in utilisation of ICSI, with the largest relative increase in non‐male factor infertility as an indication [12, 20].

We observed a marked reduction in the overall number of assisted reproduction cycles (ICSI and conventional IVF) in 2009, with the number of cycles only returning to pre‐2009 rates by 2012. It is likely that this occurred due to a variety of socio‐economic factors, including the 2008 Global Financial Crisis, which caused a four‐year plateau in the use of fertility treatment in the United States [23], local Medicare funding concerns, legislative changes and an international shortage of cycle reagent.

Our findings suggest a growing preference for empiric use of ICSI over conventional IVF. In each calendar year of our study, there was no clear indication reported for the use of ICSI for at least 34.0% of ICSI cycles. Alongside the increased proportion of non‐male factor indications for ICSI, the increase in non‐indicated ICSI is in keeping with the findings of a 2015 study that assessed indications for, and uptake of, ICSI in the American setting [12]. It is possible in some settings that this preference is driven by financial incentives [24] although this is not currently the case in Australia.

Although testicular sperm retrieval as an indication for ICSI remained stable over time, male factor infertility as an indication declined, which may reflect changes in the determination of semen parameters and data reporting, in which fewer males met the criteria for male factor infertility [25]. Donor sperm as an indication for ICSI increased over time, as access to fertility treatment improved for same‐sex couples and single women. IVF only became available to these patients in Victoria from the early 2000s onward. Parameters relating to donor sperm quality which may necessitate the need for ICSI as opposed to IVF in this setting were not available from existing datasets.

With more women seeking fertility preservation through oocyte vitrification, it was important that our study was able to examine vitrified oocyte thaw cycles as an indication for ICSI [26]. Vitrified oocyte cycles were first available in Victoria from 2008 and have since increased dramatically in their uptake [27]. Given that a vitrified oocyte thaw cycle is an absolute indication for ICSI, it is likely that the use of ICSI over conventional IVF has continued to rise in response to the increase in oocyte vitrification. The impact of these changes requires ongoing examination.

ICSI utilisation has increased in Australia, despite some evidence that ICSI entails an increased risk of birth defects [18] and autism [28], when compared with conventional IVF.

Recent studies have indicated that IVF and spontaneously conceived children have equivalent school‐age development and education outcomes [22]. Comparable studies examining the childhood impact of ICSI are very limited. While recent data suggest that ICSI and IVF conceived children have equivalent early developmental outcomes [29], the long‐term childhood impacts need to be stringently evaluated, given the increasing use of ICSI in non‐essential cases.

Our study is strengthened by the use of state‐wide data, derived from the three major fertility providers in Victoria, which allowed for a detailed examination of statewide trends. Unlike other comparable publications, our ability to examine several indications for the use of ICSI among the study cohort, including vitrified oocyte thaw cycles and donor sperm, is novel [12].

Consistent with other observational studies, we found that a lack of data completeness hampered our analysis. There are no standardised templates for assessing indications for ICSI, and uptake relies heavily on individual clinician discretion. Coupled with a lack of standardised reporting among different IVF facilities and inconsistent recording of subfertility diagnoses, the indication for ICSI had to be indirectly assessed in some cases using multiple variables and free‐text fields. Absolute indications such as surgically acquired sperm, severe male factor infertility, and vitrified oocyte thaw cycles were expected to be accurately recorded. However, the absence of individual semen analysis results, and a lack of consistency across IVF laboratories globally in the diagnosis of ‘abnormal semen parameters’ limited our ability to stratify male factor infertility indications into categories such as oligoasthenoteratozoospermia and total motile sperm count < 2 × 10^6^. Unexplained subfertility accounted for up to 0.4% of ICSI cases per year, far less than the estimated 15%–30% [30] and likely reflects incomplete data entry. Improved data collection and reporting standards may have reduced the proportion of cycles with an ‘unspecified’ indication.

Our study population included only assisted reproduction cycles which resulted in a birth after 20 weeks' gestation since there are no Victorian statewide data documenting early pregnancy loss, including ectopic pregnancy and miscarriage—factors that may influence overall rates of ICSI uptake. All IVF research would benefit from the examination of every assisted reproduction cycle, and all pregnancy outcomes—information that is not currently universally recorded.

In summary, between 2005 and 2017, there was an 18.9% increase in the proportion of assisted reproduction conceptions using ICSI over conventional IVF in Victoria. Despite the possible risk of adverse reproductive outcomes and congenital abnormalities, there was an increase in the non‐essential use of ICSI for non‐male factor infertility during the study period. Further research is warranted to examine pregnancy, birth and long‐term childhood outcomes for children conceived via ICSI in both an Australian and global setting.

## Conflicts of Interest

The authors of this manuscript have the following competing interests: B.J.V. has a paid role as a member of the Therapeutic Goods Administration. B.J.V., F.A. and C.J.S. own shares in respective IVF companies (Monash IVF, Virtus Health and Melbourne IVF).

## Supporting information


**Appendix S1:** ajo70070‐sup‐0001‐AppendixS1.docx.

## Data Availability

Data for this study was provided by various data custodians and linked by the Centre for Victorian Data linkage (https://www.health.vic.gov.au/reporting‐planning‐data/the‐centre‐for‐victorian‐data‐linkage). With relevant ethical approval, data are available upon request to the governing data custodians.

## References

[1] G. Palermo , H. Joris , P. Devroey , and A. C. Van Steirteghem , “Pregnancies After Intracytoplasmic Injection of Single Spermatozoon Into an Oocyte,” Lancet 340, no. 8810 (1992): 17–18. 1351601 10.1016/0140-6736(92)92425-f

[2] A. Van Peperstraten , M. L. Proctor , N. P. Johnson , and G. Philipson , “Techniques for Surgical Retrieval of Sperm Prior to ICSI for Azoospermia,” Cochrane Database of Systematic Reviews 19, no. 3 (2006): Cd002807. 10.1002/14651858.CD002807.pub2 16855991

[3] C. A. Benadiva , J. Nulsen , L. Siano , J. Jennings , H. B. Givargis , and D. Maier , “Intracytoplasmic Sperm Injection Overcomes Previous Fertilization Failure With Conventional In Vitro Fertilization,” Fertility and Sterility 72, no. 6 (1999): 1041–1044. 10593378 10.1016/s0015-0282(99)00403-3

[4] Pacific Fertility Center , ICSI Intracytoplasmic Sperm Injection (ICSI) (Pacific Fertility Center, 2023), https://www.pacificfertilitycenter.com/treatment‐care/male‐infertility‐treatment/icsi#:~:text=For%20those%20who%20have%20had,frozen%20(or%20vitrified)%20eggs.

[5] M. Luna , C. Bigelow , M. Duke , et al., “Should ICSI Be Recommended Routinely in Patients With Four or Fewer Oocytes Retrieved?,” Journal of Assisted Reproduction and Genetics 28, no. 10 (2011): 911–915. 21792665 10.1007/s10815-011-9614-9 PMC3220447

[6] A. R. Thornhill , C. E. deDie‐Smulders , J. P. Geraedts , et al., “ESHRE PGD Consortium ‘Best Practice Guidelines for Clinical Preimplantation Genetic Diagnosis (PGD) and Preimplantation Genetic Screening (PGS)’,” Human Reproduction 20, no. 1 (2005): 35–48. 15539444 10.1093/humrep/deh579

[7] V. Söderström‐Anttila , S. Mäkinen , T. Tuuri , and A. M. Suikkari , “Favourable Pregnancy Results With Insemination of In Vitro Matured Oocytes From Unstimulated Patients,” Human Reproduction 20, no. 6 (2005): 1534–1540. 15695312 10.1093/humrep/deh768

[8] L. N. Johnson , I. E. Sasson , M. D. Sammel , and A. Dokras , “Does Intracytoplasmic Sperm Injection Improve the Fertilization Rate and Decrease the Total Fertilization Failure Rate in Couples With Well‐Defined Unexplained Infertility? A Systematic Review and Meta‐Analysis,” Fertility and Sterility 100, no. 3 (2013): 704–711. 23773312 10.1016/j.fertnstert.2013.04.038

[9] American Society for Reproductive Medicine and Society for Assisted Reproductive Technology , “Intracytoplasmic Sperm Injection (ICSI) for Non‐Male Factor Indications: A Committee Opinion,” Fertility and Sterility 114, no. 2 (2020): 239–245. 32654822 10.1016/j.fertnstert.2020.05.032

[10] S. Tannus , W. Y. Son , A. Gilman , G. Younes , T. Shavit , and M. H. Dahan , “The Role of Intracytoplasmic Sperm Injection in Non‐Male Factor Infertility in Advanced Maternal Age,” Human Reproduction 32, no. 1 (2017): 119–124. 27852688 10.1093/humrep/dew298

[11] J. Ohl , M. Partisani , C. Wittemer , et al., “Assisted Reproduction Techniques for HIV Serodiscordant Couples: 18 Months of Experience,” Human Reproduction 18, no. 6 (2003): 1244–1249. 12773453 10.1093/humrep/deg258

[12] S. L. Boulet , A. Mehta , D. M. Kissin , L. Warner , J. F. Kawwass , and D. J. Jamieson , “Trends in Use of and Reproductive Outcomes Associated With Intracytoplasmic Sperm Injection,” Journal of the American Medical Association 313, no. 3 (2015): 255–263. 25602996 10.1001/jama.2014.17985 PMC4343214

[13] T. Jain and R. S. Gupta , “Trends in the Use of Intracytoplasmic Sperm Injection in the United States,” New England Journal of Medicine 357, no. 3 (2007): 251–257. 17634460 10.1056/NEJMsa070707

[14] Z. Li , A. Y. Wang , M. Bowman , et al., “ICSI Does Not Increase the Cumulative Live Birth Rate in Non‐Male Factor Infertility,” Human Reproduction 33, no. 7 (2018): 1322–1330. 29897449 10.1093/humrep/dey118

[15] K. Sustar , G. Rozen , F. Agresta , and A. Polyakov , “Use of Intracytoplasmic Sperm Injection (ICSI) in Normospermic Men May Result in Lower Clinical Pregnancy and Live Birth Rates,” Australian and New Zealand Journal of Obstetrics and Gynaecology 59, no. 5 (2019): 706–711. 31187499 10.1111/ajo.13004

[16] E. Cutting , F. Horta , V. Dang , M. M. van Rumste , and B. W. J. Mol , “Intracytoplasmic Sperm Injection Versus Conventional In Vitro Fertilisation in Couples With Males Presenting With Normal Total Sperm Count and Motility,” Cochrane Database of Systematic Reviews 8, no. 8 (2023): Cd001301. 37581383 10.1002/14651858.CD001301.pub2 PMC10426261

[17] M. J. Davies , V. M. Moore , K. J. Willson , et al., “Reproductive Technologies and the Risk of Birth Defects,” New England Journal of Medicine 366, no. 19 (2012): 1803–1813. 22559061 10.1056/NEJMoa1008095

[18] Y. Wang , R. Li , R. Yang , et al., “Intracytoplasmic Sperm Injection Versus Conventional In‐Vitro Fertilisation for Couples With Infertility With Non‐Severe Male Factor: A Multicentre, Open‐Label, Randomised Controlled Trial,” Lancet 403 (2024): 924–934, 10.1016/S0140-6736(23)02416-9. 38330980

[19] C. Wyns , C. De Geyter , C. Calhaz‐Jorge , et al., “ART in Europe, 2018: Results Generated From European Registries by ESHRE,” Human Reproduction Open 2022, no. 3 (2022): hoac022, 10.1093/hropen/hoac022. 35795850 PMC9252765

[20] A. Nyboe Andersen , E. Carlsen , and A. Loft , “Trends in the Use of Intracytoplasmatic Sperm Injection Marked Variability Between Countries,” Human Reproduction Update 14, no. 6 (2008): 593–604. 18708651 10.1093/humupd/dmn032

[21] A. P. Ferraretti and L. Gianaroli , “The Bologna Criteria for the Definition of Poor Ovarian Responders: Is There a Need for Revision?,” Human Reproduction 29, no. 9 (2014): 1842–1845. 25008235 10.1093/humrep/deu139

[22] A. L. Kennedy , B. J. Vollenhoven , R. J. Hiscock , et al., “School‐Age Outcomes Among IVF‐Conceived Children: A Population‐Wide Cohort Study,” PLoS Medicine 20, no. 1 (2023): e1004148. 36693021 10.1371/journal.pmed.1004148 PMC9873192

[23] P. S. Gromski , A. Smith , D. A. Lawlor , F. I. Sharara , and S. M. Nelson , “2008 Financial Crisis Versus 2020 Economic Fallout: How COVID‐19 Might Influence Fertility Treatment and Live Births,” Reproductive Biomedicine Online 42, no. 6 (2021): 1087–1096. 33931369 10.1016/j.rbmo.2021.03.017

[24] P. Zagadailov , K. S. Cho , and D. B. Seifer , “Differences in ICSI Utilization Rates Among States With Insurance Mandates for ART Coverage,” Reproductive Biology and Endocrinology 19 (2021): 174, 10.1186/s12958-021-00856-4. 34847941 PMC8630859

[25] K. S. Murray , A. James , J. B. McGeady , M. L. Reed , W. W. Kuang , and A. K. Nangia , “The Effect of the New 2010 World Health Organization Criteria for Semen Analyses on Male Infertility,” Fertility and Sterility 98, no. 6 (2012): 1428–1431. 22921910 10.1016/j.fertnstert.2012.07.1130

[26] D. Lu , “‘Very Pragmatic’: 42% of Australian Women Are Open to Egg Freezing as a Work Perk,” The Guardian, (2021), https://www.theguardian.com/science/2021/jul/13/a‐very‐pragmatic‐decision‐42‐of‐australian‐women‐are‐open‐to‐egg‐freezing‐as‐a‐work‐perk#:~:text=The%20study%2C%20which%20surveyed%20656,were%20opposed%20to%20the%20idea.

[27] Victoria Assisted Reproductive Treatment Authority (VARTA) , “VARTA Annual Report 2022,” (2022), https://www.varta.org.au/resources/annual‐reports.

[28] S. Sandin , K. G. Nygren , A. Iliadou , C. M. Hultman , and A. Reichenberg , “Autism and Mental Retardation Among Offspring Born After In Vitro Fertilization,” Journal of the American Medical Association 310, no. 1 (2013): 75–84. 23821091 10.1001/jama.2013.7222

[29] A. L. Kennedy , R. J. Hiscock , B. J. Vollenhoven , et al., “School‐Age Outcomes Among IVF and ICSI‐Conceived Children: A Causal Inference Analysis Using Linked Population‐Wide Data,” BMC Medicine 23 (2025): 194. 40170012 10.1186/s12916-025-03963-w PMC11963277

[30] A. Quaas and A. Dokras , “Diagnosis and Treatment of Unexplained Infertility,” Reviews in Obstetrics and Gynecology 1, no. 2 (2008): 69–76. 18769664 PMC2505167

