# Supplementary material for: Trends in the Use and Indications for Intracytoplasmic Sperm Injection Between 2005 and 2017: A State‐Wide Descriptive Cohort Analysis
Source: Aust N Z J Obstet Gynaecol. 2025 Nov 5;66(1):e70070. doi: 10.1111/ajo.70070 (PMC12881710; doi:10.1111/ajo.70070)

# Supplementary Materials

**Table S1:** annual frequency of conventional IVF, ICSI, and total cycles, 2005-2017.

|  | **IVF, n (%)** | **ICSI, n (%)** | **Total, n (%)** |
| --- | --- | --- | --- |
| **Year** |  |  |  |
|  | **N = 9,229** | **N = 22,873** | **N = 32,102** |
| 2005 | 770 (39.4) | 1,182 (60.6) | 1,952 |
| 2006 | 839 (39.4) | 1,288 (60.6) | 2,127 |
| 2007 | 931 (40.0) | 1,399 (60.0) | 2,330 |
| 2008 | 963 (40.1) | 1,441 (59.9) | 2,404 |
| 2009 | 685 (36.1) | 1,213 (63.9) | 1,898 |
| 2010 | 669 (30.2) | 1,548 (69.8) | 2,217 |
| 2011 | 690 (29.7) | 1,635 (70.3) | 2,325 |
| 2012 | 680 (24.9) | 2,048 (75.1) | 2,728 |
| 2013 | 640 (24.0) | 2,022 (76.0) | 2,662 |
| 2014 | 628 (22.1) | 2,220 (77.9) | 2,848 |
| 2015 | 591 (20.9) | 2,240 (79.1) | 2,831 |
| 2016 | 540 (19.1) | 2,293 (80.9) | 2,833 |
| 2017 | 603 (20.5) | 2,344 (79.5) | 2,947 |

IVF = in vitro fertilisation, ICSI = intracytoplasmic sperm injection.

**Table S2:** annual frequency of ICSI cycles stratified by indication, 2005-2017.

# n (%) n (%) n (%) n (%) n (%) n (%) n (%) n (%) n (%) N

**Year**

**Total**

**Testicular Sperm**

**Retrieval**

**Male Factor**

**Infertility**

**Vitrified Oocyte**

**Thaw Cycle**

**Donor Sperm**

**Female Factors**

**Associated with**

**Poor IVF Advanced**

**Maternal Age**

**Pre-implantation**

**Genetic Testing**

**Unexplained**

**Subfertility**

**Unspecified**

2005

93 (7.9)

503 (42.6)

1 (0.1)

42 (3.6)

73 (6.2)

28 (2.4)

13 (1.1)

0 (0.0)

429 (36.3)

1,182

2006 102 (7.9) 534 (41.5) 0 (0.0) 66 (5.1) 81 (6.3) 35 (2.7) 10 (0.8) 0 (0.0) 460 (35.7) 1,288

2007

104 (7.4) 579 (41.4)

3 (0.2)

69 (4.9)

91 (6.5)

38 (2.7)

21 (1.5)

1 (0.1)

493 (35.2)

1,399

2008 121 (8.4) 517 (35.9) 0 (0.0) 55 (3.8) 110 (7.6) 51 (3.5) 13 (0.9) 1 (0.1) 573 (39.8) 1,441

2009

133 (11.0) 228 (18.8)

0 (0.0)

77 (6.3)

175 (14.4)

89 (7.3)

2 (0.2)

1 (0.1)

508 (41.9)

1,213

2010 134 (8.7) 230 (14.9) 2 (0.1) 139 (9.0) 211 (13.6) 106 (6.8) 14 (0.9) 2 (0.1) 710 (45.9) 1,548

2011

145 (8.9) 279 (17.1)

4 (0.2)

136 (8.3) 213 (13.0)

95 (5.8)

33 (2.0)

7 (0.4)

723 (44.2)

1,635

2012 157 (7.7) 393 (19.2) 7 (0.3) 181 (8.8) 263 (12.8) 117 (5.7) 81 (4.0) 9 (0.4) 840 (41.0) 2,048

| 2013 | 157 (7.8) | 365 (18.1) | 6 (0.3) | 191 (9.4) | 247 (12.2) | 121 (6.0) | 73 (3.6) | 3 (0.1) | 859 (42.5) | 2,022 |
| --- | --- | --- | --- | --- | --- | --- | --- | --- | --- | --- |
| 2014 | 187 (8.4) | 382 (17.2) | 22 (1.0) | 183 (8.2) | 309 (13.9) | 112 (5.0) | 87 (3.9) | 4 (0.2) | 934 (42.1) | 2,220 |
| 2015 | 173 (7.7) | 390 (17.4) | 25 (1.1) | 213 (9.5) | 278 (12.4) | 134 (6.0) | 140 (6.3) | 8 (0.4) | 879 (39.2) | 2,240 |
| 2016 | 152 (6.6) | 332 (14.5) | 39 (1.7) | 217 (9.5) | 289 (12.6) | 139 (6.1) | 317 (13.8) | 3 (0.1) | 805 (35.1) | 2,293 |
| 2017 | 156 (6.7) | 362 (15.4) | 24 (1.0) | 255 (10.9) | 251 (10.7) | 140 (6.0) | 344 (14.7) | 3 (0.1) | 809 (34.5) | 2,344 |

ICSI = intracytoplasmic sperm injection, IVF = in vitro fertilisation.

Testicular sperm retrieval: ptrend = 0.153; male factor infertility: ptrend = 0.007; vitrified oocyte thaw cycle: ptrend = 0.016; donor sperm: ptrend = 0.001; female factors associated with poor IVF outcomes: ptrend = 0.005; advanced maternal age: ptrend = 0.005; pre-implantation genetic testing: ptrend = 0.004; unexplained subfertility: ptrend = 0.304, unspecified: ptrend = 0.015.

**Figure S1:** area chart displaying percentage frequency of conventional IVF compared to ICSI cycles, 2005-2017.

IVF = in vitro fertilisation, ICSI = intracytoplasmic sperm injection.


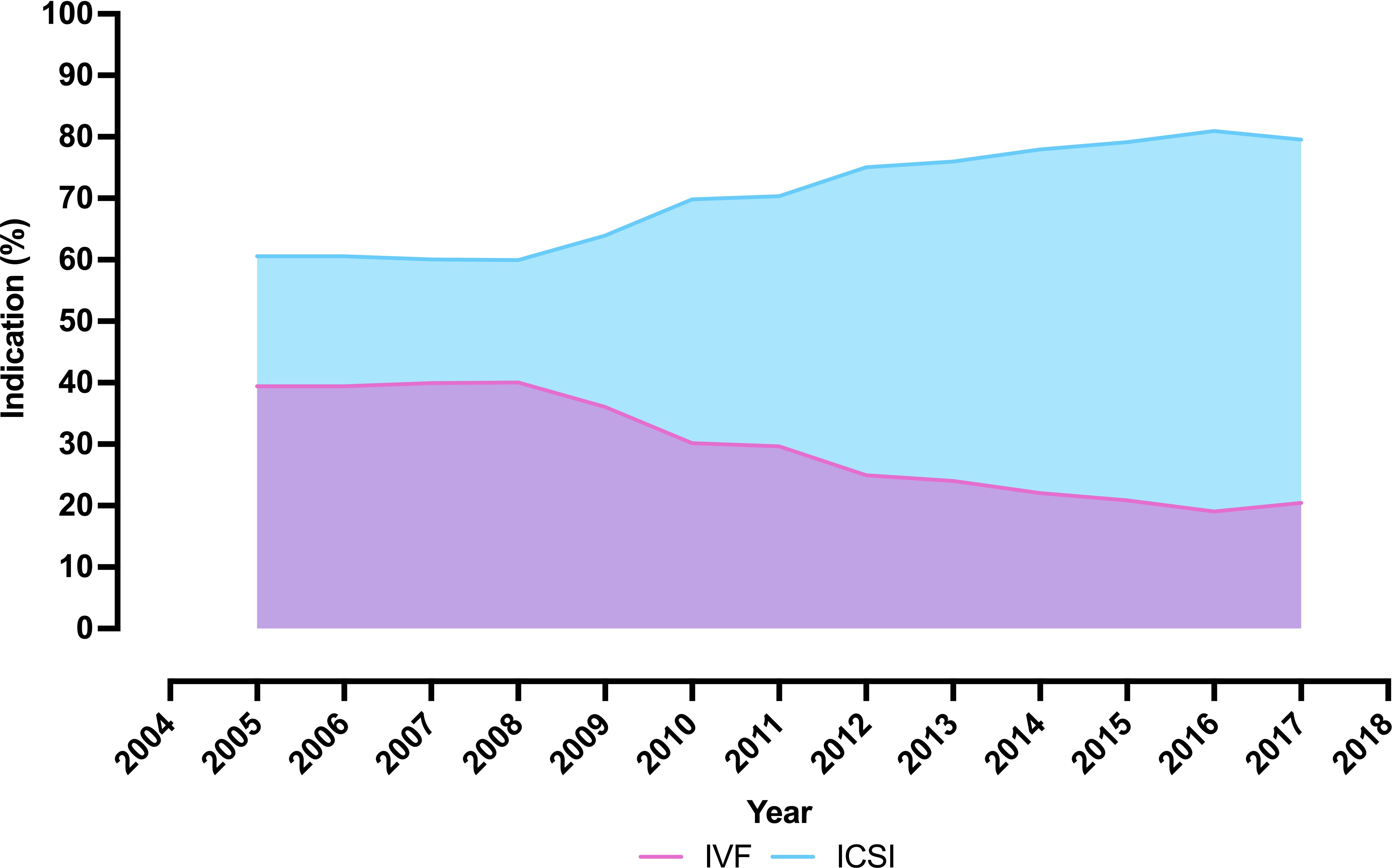


**Figure S2:** line chart displaying annual number of ICSI cycles, stratified by indication for ICSI, 2005-2017.

ICSI = intracytoplasmic sperm injection, IVF = in vitro fertilisation.

Testicular sperm retrieval: ptrend = 0.15; male factor infertility: ptrend = 0.007; vitrified oocyte thaw cycle: ptrend = 0.016; donor sperm: ptrend = 0.001; female factors associated with poor IVF outcomes: ptrend = 0.005; advanced maternal age: ptrend = 0.005; pre-implantation genetic testing: ptrend = 0.004; unexplained subfertility: ptrend = 0.30, unspecified: ptrend = 0.015.


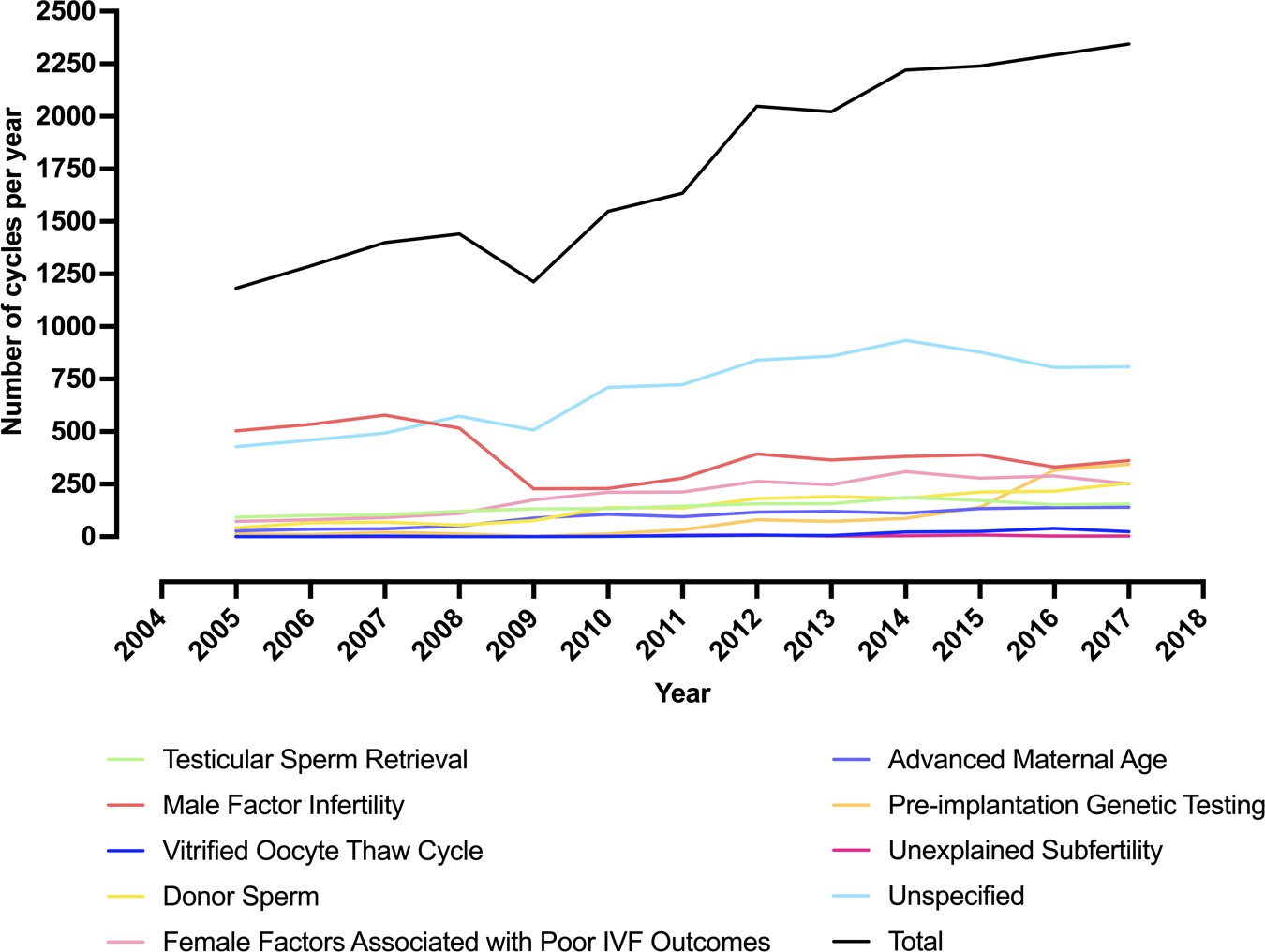

Supplement: Supplementary file 1 — Appendix S1: ajo70070‐sup‐0001‐AppendixS1.docx. [file AJO-66-0-s001.docx]
